# Phenotypic Characterization of *pilA, pilB,* and *pilD* Mutants of *Acinetobacter baumannii* 5075: Impacts on Growth, Biofilm Formation, and Tazobactam Response

**DOI:** 10.3390/antibiotics14080816

**Published:** 2025-08-09

**Authors:** Joel H. Salinas, Fatma Pinar Gordesli-Duatepe, Angelica Diaz-Sanchez, Nehal I. Abu-Lail

**Affiliations:** 1 Department of Biomedical Engineering and Chemical Engineering, University of Texas at San Antonio, San Antonio, TX 78249, USA; joel.salinas@my.utsa.edu (J.H.S.J.); angelica.diaz-sanchez@my.utsa.edu (A.D.-S.); 2 Department of Genetics and Bioengineering, Izmir University of Economics, Izmir 35330, Türkiye; pinar.gordesli@ieu.edu.tr

**Keywords:** *Acinetobacter baumannii* 5075, biofilm, growth kinetics, tazobactam (TAZ), time–kill assay, Type IV pili

## Abstract

Background/Objectives: The Type IV pilus assembly system in *Acinetobacter baumannii* is a major determinant of its pathogenicity, playing a role in surface-associated functions via the biogenesis of Type IV pili (T4P). Tazobactam (TAZ) is a well-characterized β-lactamase inhibitor, primarily used in combination with β-lactam antibiotics such as piperacillin (PIP) to counteract bacterial resistance mechanisms. While *A. baumannii* resistance to β-lactam antibiotics has been well studied, the influence of T4P on its susceptibility to TAZ remains largely unexplored. For this reason, we investigated how multidrug-resistant *A. baumannii* 5075 (AB5075) responds to TAZ by assessing the roles of *pilA*, *pilB*, and *pilD* in bacterial growth and biofilm formation under direct TAZ exposure, with a focus on phenotypic characterization rather than molecular mechanisms. Methods: Bacterial growth kinetics were quantified by measuring the optical densities of cell suspensions and the colony forming units per volume (CFUs/mL) at different time intervals. Time-kill assays and microtiter dish biofilm formation assays were used to evaluate how effectively TAZ can inhibit growth and biofilm formation, respectively. Results: Time–kill assays confirmed that 32 µg/mL of TAZ inhibited growth in both wild-type (WT) and mutant strains, with the *pilD* mutant showing initial resistance before eventual inhibition. Biofilm assays showed that the *pilA* mutant had the highest biofilm formation at 8 h, surpassing the WT strain. A prolonged 32 µg/mL of TAZ exposure (24–36 h) significantly reduced biofilm production across all strains, with inhibition rates reaching 89% for the WT, 82% for the *pilA* mutant, 91% for the *pilB* mutant, and 86% for the *pilD* mutant. Conclusion: These findings deepen our understanding of the strain-specific roles of T4P components in growth and biofilm regulation in AB5075, and highlight the potential of TAZ as a therapeutic strategy against biofilm-associated infections.

## 1. Introduction

The ESKAPE acronym, designated by the Infectious Diseases Society of America (IDSA), encompasses the six highly virulent and resistant bacterial genera—*Enterococcus faecium*, *Staphylococcus aureus*, *Klebsiella pneumoniae*, *Acinetobacter baumannii*, *Pseudomonas aeruginosa*, and *Enterobacter* spp.—which collectively account for the majority of hospital-acquired infections and are capable of evading the effects of most clinically available antibiotics [1,2]. Among the ESKAPE group, the Gram-negative *A. baumannii* poses a particularly severe clinical threat, with reported mortality rates reaching up to 44% in elderly hospitalized patients and 45–60% in Intensive Care Units (ICUs) [3,4,5]. Its persistence on abiotic surfaces and efficient transmission in healthcare settings are key factors contributing to its success as a nosocomial pathogen [5,6,7,8]. In recognition of its growing clinical burden, the Centers for Disease Control and Prevention (CDC) has classified *A. baumannii* as a serious public health threat due to its resistance to many β-lactam antibiotics [8,9]. The widespread use of antibiotics, combined with inadequate infection control, has facilitated the evolution of resistant *A. baumannii* strains, with genetic investigations revealing numerous genes encoding β-lactamases and outer membrane proteins that promote antibiotic resistance [8,10,11]. Understanding these mechanisms is critical for the development of effective therapeutic strategies, especially in the face of emerging resistance among *A. baumannii* and other ESKAPE pathogens.

Among the various virulence factors that facilitate the pathogenicity of Gram-negative bacteria, the Type IV pilus assembly system plays a pivotal role in surface adhesion, biofilm formation, twitching motility, host interactions, and natural transformation [12,13,14]. This system drives the biogenesis of Type IV pili (T4P)—long and highly dynamic filamentous appendages assembled from pilin subunits into helical fibers and capable of rapid surface extension and retraction [12,13,14]. The disruption of pilus assembly in numerous Gram-negative pathogens significantly impairs their virulence, emphasizing the important contribution of pili to their pathogenic potential [12]. These filamentous appendages form the basis of *Acinetobacter* virulence as well as their ability to acquire and integrate genetic material from the environment [13]. Consequently, this leads to the rapid dissemination of antibiotic resistance genes, significantly increasing the challenges associated with bacterial treatment. Unlike other resistance determinants—such as β-lactamases, outer membrane proteins (OMPs), efflux pumps, and penicillin-binding proteins (PBPs)—T4P structures can also contribute to antibiotic tolerance via biofilm formation [15]. Given the strong association between biofilm formation, chronic infections, the uptake and integration of environmental genetic material, and reduced treatment efficacy, uncovering the roles of genetic and structural components of the T4P machinery on bacterial growth and biofilm formation is of increasing importance for the development of alternative antimicrobial strategies.

In response to increasing β-lactam resistance in *A. baumannii*, and as an alternative to first-line treatment options such as β-lactam/β-lactamase inhibitor combinations (e.g., piperacillin (PIP)/tazobactam (TAZ) for susceptible strains), evidence that some β-lactamase inhibitors—such as TAZ—may exhibit limited but potentially relevant intrinsic antibacterial activity when used alone [16,17], independent of their traditional role in combination therapy, suggests that their standalone efficacy warrants further investigation. Although TAZ generally displays low intrinsic activity against most Gram-positive and Gram-negative bacteria, exceptions such as *Escherichia coli*, *Bacteroides fragilis*, and *Acinetobacter* spp. point to a more complex mechanism of action [16,17,18,19]. Its antibacterial effect has been linked to binding penicillin-binding protein 2 (PBP2), which disrupts cell wall synthesis and alters cell morphology [18,19,20]. In *E. coli*, for example, TAZ has been shown to bind PBP2 and induce rapid lysis of spheroplasts at relatively high concentrations (MIC: 512 µg/mL) [18,19]. Moreover, its measurable activity against *B. fragilis* (MIC: 4 to 8 µg/mL) [19], along with its potential interaction with *Acinetobacter* spp. (MIC: 4 to 51 µg/mL) [16,17], raises the question of whether TAZ alone may offer therapeutic benefit against certain *A. baumannii* strains. These findings support the idea that TAZ may exert biologically relevant effects beyond β-lactamase inhibition. Consistent with this notion, a recent genome-wide transposon mutagenesis study demonstrated that exposure of *E. coli* and *K. pneumoniae* to TAZ selected for efflux pump and cell envelope mutants associated with multidrug resistance [21]. In the same study, additional pathways, such as those involving the shikimate kinase AroK, were also implicated in TAZ susceptibility. These results indicate that TAZ can impose selective pressure even in the absence of a β-lactam partner and modulate bacterial physiology through mechanisms unrelated to enzyme inhibition, thereby further reinforcing the rationale for investigating its standalone effects in clinically relevant *A. baumannii* strains.

Given the multifaceted roles of T4P structures in surface-associated processes and virulence, including their potential involvement in biofilm-mediated tolerance, disruption of Type IV pilus assembly system components could alter the physiological state of the cell and thereby modulate its response to external stressors, including antimicrobial agents such as TAZ. Our study investigates the roles of *pilA*, *pilB*, and *pilD* in the growth, biofilm formation, and TAZ response of *A. baumannii*, with a focus on phenotypic characterization rather than molecular mechanisms. The aim is to identify observable differences associated with pilus gene disruptions that may serve as a basis for future mechanistic studies. To this end, we investigated the contribution of T4P to the response of *A. baumannii* to TAZ using the well-characterized and highly virulent *A. baumannii* 5075 (AB5075) strain [4] and its pili-deficient mutants (*pilA, pilB*, and *pilD* mutants). AB5075 is a multidrug-resistant strain originally isolated in 2008 from a combat-related wound infection (osteomyelitis of the tibia) at Walter Reed Army Medical Center, and infections with this strain were shown to be considerably more severe than those caused by other clinical isolates, as reflected by significantly decreased survival rates in animal models [4]. It belongs to global clone 1 and is considered a clinically relevant and genetically tractable model [4,22]. Furthermore, AB5075 exhibits elevated resistance to multiple antibiotic classes, including carbapenems, aminoglycosides, polymyxins, and extended-spectrum β-lactams, such as those conferred by the Oxa-23 β-lactamase [23]. These features make AB5075 a highly suitable model for examining antibiotic response phenotypes, including the less commonly studied effects of TAZ monotherapy.

We systematically evaluated the bacterial growth kinetics, colony-forming ability, biofilm development, and susceptibility to TAZ in the AB5075 strain and its pili-deficient mutants, in order to assess the phenotypic consequences of disrupting key T4P components under defined experimental conditions.

## 2. Results and Discussion

### 2.1. Growth Kinetics of the Planktonic AB5075 Strain and Its Mutants

**Figure 1:**
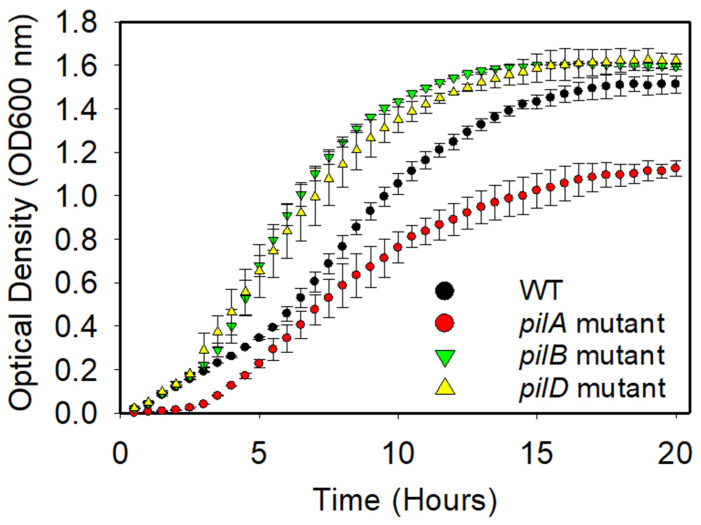
Average growth kinetics of the AB5075 strain and its investigated mutants, with each consisting of a biological triplicate and each being denoted with a unique color and symbol. Error bars represent the calculated standard deviation from a triplicate. Pairwise multiple comparisons (Dunn’s test) identified the *pilA* mutant as significantly different from both the *pilB* mutant (*p* < 0.001) and *pilD* mutant (*p* < 0.001).

Figure 1 demonstrates the growth kinetics of the AB5075 strain and its tested mutants. As is typical with bacteria, a period of exponential growth can be observed during the early hours of incubation, followed by a stationary phase that begins around 10 h and continues up to 20 h. To more precisely define growth phases, a natural logarithm transformation of the optical density values (OD600 nm) was performed. Based on the resulting ln(OD) vs. time plots (Figure S1A,B in the Supplementary Material), the intervals between 2 and 5 h and 5 and 8 h were approximated as representing the early and mid-exponential phases, respectively. Specific growth constants and doubling times were computed using linear regressions over these intervals and are presented in Table 1. The main goal of this experiment was to establish a basis for future research by allowing comparisons with existing findings. The data obtained serves as a fundamental reference point for understanding how mutations in *pilA*, *pilB*, and *pilD* affect the growth kinetics of *A. baumannii*. Moreover, it emphasizes the significance of thoroughly characterizing mutant strains to gain insight into the influence of specific genetic changes on bacterial growth and adaptation.

Although initial comparisons using the rank sum test indicated statistically significant differences in the optical density data obtained for the mutant strains in comparison to the data obtained for the wild-type (WT) strain, specifically for the *pilA* mutant (*p* = 0.007), *pilB* mutant (*p* = 0.007), and *pilD* mutant (*p* = 0.029), these differences did not remain significant after adjustment for pairwise multiple comparisons using Dunn’s test. However, importantly, Dunn’s test identified the *pilA* mutant as significantly different from both the *pilB* (*p* < 0.001) and *pilD* (*p* < 0.001) mutants, suggesting that the *pilA* mutant exhibits a distinct phenotype compared to the other mutants.

As shown in Table 1, during the early exponential growth phase (2–5 h), the quantified specific growth constants for all mutant strains were higher than that of the WT strain. In the mid-exponential phase (5–8 h), only the *pilA* mutant maintained an increased growth rate compared to the WT and the other mutants. While these trends are biologically notable, statistical comparisons across all groups yielded significance only for the *pilA* mutant relative to both the *pilB* and *pilD* mutants (*p* < 0.001), as mentioned above, potentially due to intra-group variability and the inherent limitations of pairwise non-parametric tests. Nevertheless, the consistent pattern of increased growth rate observed for the *pilA* mutant (Table 1) may reflect biologically meaningful differences that warrant further investigation.

Understanding how PilA, PilB, and PilD expression is regulated within the cell may provide further insights into their effects in bacterial growth kinetics. The Type IV pilus consists of a major pilin subunit, PilA, and various minor pilins, which differ among bacterial species [24]. Pilus extension and retraction are driven by the cytosolic PilB and PilT/PilU ATPases, respectively [24,25]. PilD functions as a prepilin peptidase that processes pilin subunits prior to their incorporation into the pilus structure [24,25,26]. In the absence of PilA, the major pilin subunit, the assembly of functional pili is impaired, which may reduce the cellular energy demand associated with pilus formation. This reduction in energy expenditure could allow for the reallocation of resources toward other cellular processes, such as an increased growth rate. Previously, coarse-grained mechanistic models have shown that bacteria operate under strict limitations in energy, ribosome availability, and proteome capacity, and that the downregulation of energetically costly systems can free up resources for translation and biomass accumulation [27,28]. Experimental data support this view: nonpiliated *Neisseria gonorrhoeae* variants exhibit higher expression of ribosomal and translational genes and an increased growth rate compared to piliated strains [29], while range expansion experiments show that nonpiliated cells can spatially outcompete piliated ones, benefiting from greater access to nutrients [30]. Although our study did not assess intracellular resource usage or energy flux, the higher specific growth rate computed for the *pilA* mutant may be meaningful and could reflect a shift in biosynthetic investment away from pili-related functions.

### 2.2. Colony-Forming Abilities of the AB5075 Strain and Its Mutants

Table 2 illustrates the CFU/mL measurements for each tested strain at various time intervals. Analyzing the CFU/mL of the AB5075 WT and mutant strains in the absence of antibiotics reveals notable trends, shedding further light on their growth characteristics. Interestingly, the *pilA* mutant consistently displayed the highest CFU/mL counts across all recorded time points, indicating its robust colony formation capacity, followed by the *pilD* mutant, while the *pilB* mutant and WT displayed lower colony counts.

As discussed above, T4P biogenesis is an energy-intensive process, requiring substantial ATP investment for pilus elongation and retraction [24,25]. Similarly, colony formation is also an energy-demanding process, as it involves not only cell proliferation but also active surface behaviors such as twitching motility and the expression of adhesion factors [31,32]. Twitching motility, in particular, relies on the dynamic extension and retraction of T4P structures, which are tightly coupled to cellular energy status [32,33,34,35]. Therefore, mutations disrupting T4P components may significantly alter energy allocation within the cell, ultimately influencing both growth dynamics and surface colonization capacity on solid surfaces.

Previous studies have shown that *A. baumannii* utilizes PilA, PilD, and PilT to mediate twitching motility [25,34,36]. A positive correlation between the PilA encoding gene and the degree of twitching motility has also been demonstrated in clinical isolates [37]. The absence of *pilA* or *pilD* may allow the redirection of cellular energy toward survival or surface colonization, resulting in elevated CFU/mL counts. In the case of the *pilA* mutant, the loss of PilA likely halts pilus assembly altogether, eliminating twitching motility and possibly reducing energy expenditure, thereby favoring colony formation. In *A. baumannii*, the type II secretion system, which is responsible for assembling a pseudopilus assumed to function as a piston to facilitate protein secretion [38], shares the processing protein PilD with T4P [26]. Since PilD is responsible for processing not only T4P subunits but also other outer membrane proteins, the relatively high CFU/mL count may be attributed to compensatory upregulation of alternative adhesins or reduced cell dispersal due to motility loss.

### 2.3. Tazobactam Response of the AB5075 Strain and Its Mutants Evaluated Using Time–Kill Assays

Rising β-lactam resistance in *A. baumannii* and the limited efficacy of conventional β-lactam/β-lactamase inhibitor combinations (e.g., PIP/TAZ) against resistant strains highlight the urgent need for alternative therapeutic strategies. Although TAZ is primarily used as a β-lactamase inhibitor, it has been reported to exhibit limited but potentially relevant intrinsic antibacterial activity when used alone, warranting further investigation into its standalone efficacy. Previously, the intrinsic activity of TAZ against *Acinetobacter* spp. was reported with MIC values ranging between 4 and 51 µg/mL [16,17]. In this study, a high concentration of 32 µg/mL TAZ was selected to evaluate potential physiological responses of *A. baumannii*. This concentration aligns with interpretive frameworks presented by the Clinical and Laboratory Standards Institute (CLSI) for MIC-based susceptibility thresholds, in which ≥32 µg/mL is often designated as the resistance breakpoint [39].

The time–kill assay played a pivotal role in this study, aiming to evaluate whether a concentration of 32 µg/mL of TAZ could effectively inhibit bacterial growth over a period of approximately 40 h, with regular antibiotic refreshment every 12 h. As can be seen from Figure S2 (in the Supplementary Material), the time–kill assay based on the measured absorbance (optical density) values revealed that 32 µg/mL of TAZ effectively halted bacterial growth for the WT strain. The growth kinetics and inhibitory effects mirrored those observed in the WT strain, thus confirming the efficacy of 32 µg/mL of TAZ in inhibiting bacterial growth for all mutant strains.

The growth observed for the *pilD* mutant during the first 12 h of TAZ treatment (Figure S2) may indicate the activation of an early adaptation or persistence-like mechanism, which allowed the mutant strain to temporarily withstand the antibiotic stress. A recent study on *P. aeruginosa* clinical isolates reported a similar phenomenon, where *pilD* inactivation led to a significantly higher level of persister cell survival following ciprofloxacin treatment [40]. Persister cells are subpopulations that resist antimicrobial treatment by entering a state of dormancy or quiescence, during which they become transiently tolerant to antibiotics [41,42]. Notably, this *pilD*-mediated persistence did not rely on classical mechanisms such as phenazine pyocyanin production, biofilm formation, or the stringent response [40]. Although the species and antibiotic context differ, this finding may suggest that *pilD* inactivation can modulate bacterial persistence through alternative, yet unidentified, pathways. In our case, however, this transient resistance was not sustained: after 12 h and a replenishment of the TAZ concentration, the *pilD* mutant strain’s initial tolerance mechanisms proved insufficient against continuous TAZ exposure, ultimately leading to growth inhibition. This biphasic response requires further investigation into the potential link between the *pilD* gene and the antibiotic persistence mechanisms in *A. baumannii*.

**Figure 2:**
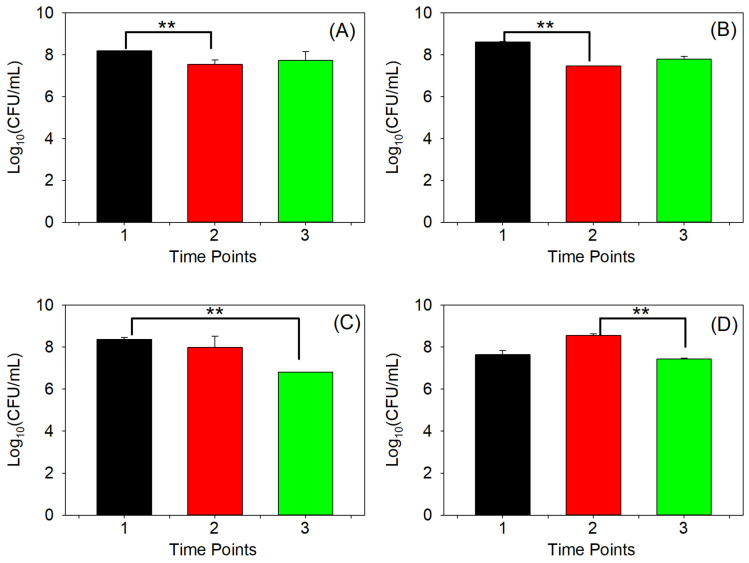
Time–kill assay based on log_10_-transformed CFU/mL measurements of the AB5075 strain and its mutants treated with 32 µg/mL TAZ, with antibiotic replenishment every 12 h. Measurements were taken at 4, 11, and 20 h, corresponding to time points 1, 2, and 3, respectively, for the (**A**) WT; (**B**) *pilA* mutant; (**C**) *pilB* mutant; and (**D**) *pilD* mutant. ** Indicates significance (*p* < 0.05).

Figure 2 presents the results of the time–kill assay based on log_10_-transformed CFU/mL measurements for all AB5075 strains treated with 32 µg/mL TAZ, with antibiotic replenishment every 12 h, assessed at three time points: 4, 11, and 20 h. (The corresponding non-transformed CFU/mL data are presented in Figure S3 in the Supplementary Material.) Statistical analysis of the data yielded several notable findings. For the WT and *pilA* mutant strains (Figure 2A,B and Figure S3A,B), ANOVA on ranks revealed significant differences at time points 1 and 2 (*p* < 0.05). In the case of the *pilB* mutant strain (Figure 2C, and Figure S3C), significant differences were observed at time points 1 and 3 (*p* < 0.05), while for the *pilD* mutant (Figure 2D, and Figure S3D), significance was found at time points 2 and 3 (*p* < 0.05). These temporal variations in statistical significance highlight fluctuations in bacterial growth dynamics and emphasize the importance of assessing multiple time points in antimicrobial efficacy evaluations. The presence of persister cells may also contribute to the observed fluctuations in CFU counts [41,42,43,44].

Despite displaying absorbance profiles similar to those of the WT and *pilB* mutant strains (Figure S2), the *pilA* mutant demonstrated the highest CFU/mL count at the 4 h mark following initial TAZ exposure (Figure 2B and Figure S3B). This finding suggests that the *pilA* mutant may activate early-phase mechanisms that enhance cellular viability under antibiotic stress. Although previous interpretations were made under antibiotic-free conditions, it is plausible that similar physiological shifts occur in the presence of TAZ. As previously discussed, the absence of PilA or PilD may redirect cellular energy away from pilus-mediated motility and toward alternative survival strategies or enhanced surface colonization—even under antibiotic pressure. At 11 h, the *pilD* mutant exhibited the highest CFU/mL count (Figure 2D and Figure S3D), consistent with its robust growth observed in absorbance data (Figure S2), suggesting the activation of an early adaptive or persistence-like response. Interestingly, by 20 h, the WT strain displayed the highest CFU/mL count (Figure 2A and Figure S3A), deviating from its corresponding absorbance trend (Figure S2). This discrepancy underscores the complexity of interpreting bacterial viability and growth, particularly under antibiotic exposure.

Overall, the complex nature of bacterial responses to antibiotics, including dynamic growth patterns and adaptive mechanisms, reflects the multifaceted challenges in interpreting CFU count variations.

### 2.4. Tazobactam Response of Biofilms Formed by the AB5075 Strain and Its Mutants at Distinct Times

**Figure 3:**
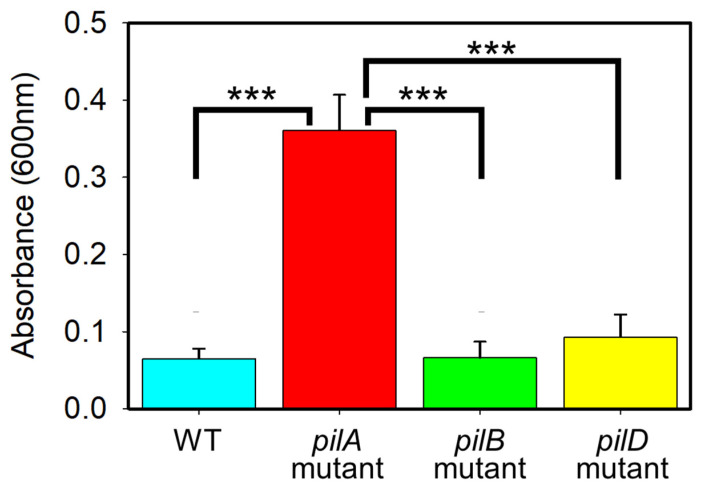
Comparison of biofilm formation by the AB5075 strain and its mutants after 8 h without antibiotic exposure. *** indicates (*p* < 0.001).

Figure 3 illustrates the biofilm formation capacities of all strains after 8 h without TAZ exposure. Pairwise comparisons of mean responses were performed using the Tukey Test. Given that the *pilA* mutant displayed an accelerated planktonic growth rate compared to the WT (Table 1), as well as the highest CFU/mL counts across all recorded time points (Table 2), it was expected that the *pilA* mutant strain would also exhibit the highest level of biofilm formation, surpassing the WT strain, as confirmed by the results in Figure 3. This enhanced biofilm production may result from the loss of twitching motility. As previously reported, PilA from AB5075 exhibits a distinct electronegative surface that favors twitching motility over biofilm formation [14], and a positive correlation exists between the *pilA* gene and the degree of twitching motility in clinical isolates of *A. baumannii* [37]. In the absence of PilA, impaired twitching motility likely redirects cellular energy and resources toward surface colonization and biomass accumulation. Moreover, since *pilA* mutants do not display post-division movement [45], this immobilization may promote the establishment of a dense and stable basal layer of adherent cells during early biofilm development. Such a stationary population may enhance the structural integrity of the biofilm and facilitate subsequent biofilm maturation.

Although the existing literature acknowledges the general significance of pilins in biofilm formation [12,13,14,15,46,47], suggesting that the biofilm-forming ability of *A. baumannii* is dependent on pili production and assembly [46,47], our findings diverge from this trend. This discrepancy may stem from strain-specific variations, as previous studies on *A. baumannii* biofilm development have largely focused on strains (e.g., ATCC 17978, ATCC 19606, and VGH1-7) other than the AB5075 strain employed in our study [46,47]. To better understand this deviation, it is useful to examine studies in *P. aeruginosa*, a model organism for T4P research, where the pilus biogenesis system is more extensively characterized. In *P. aeruginosa*, nearly 50 genes are involved in T4P regulation and assembly, indicating a complex and tightly controlled structure–function relationship [48]. However, even in this well-studied organism, the role of T4P components in biofilm development remains context-dependent. Some twitching-deficient mutants have been shown to form larger microcolonies than the wild-type strain *P. aeruginosa* PAO1 [49,50]. Notably, *P. aeruginosa* mutants lacking twitching motility exhibited surface attachment rates approximately five times greater than other strains, with their microcolonies coalescing into increasingly larger structures over time [49]. However, other studies showed that T4P mutants of *P. aeruginosa* PA14 were unable to form microcolonies [51]. One study reported that *pilA* mutants produced microcolonies similar to those of the wild-type *P. aeruginosa* strain PAO1 [52], further highlighting the variability in outcomes. These conflicting results suggest that differences in strain backgrounds and experimental setups can significantly influence the observed phenotypes of T4P-deficient mutants [49,50,52]. Thus, the enhanced biofilm formation observed in our *pilA* mutant may reflect a strain-specific adaptation in AB5075, potentially favoring sessile growth when twitching motility is lost.

**Figure 4:**
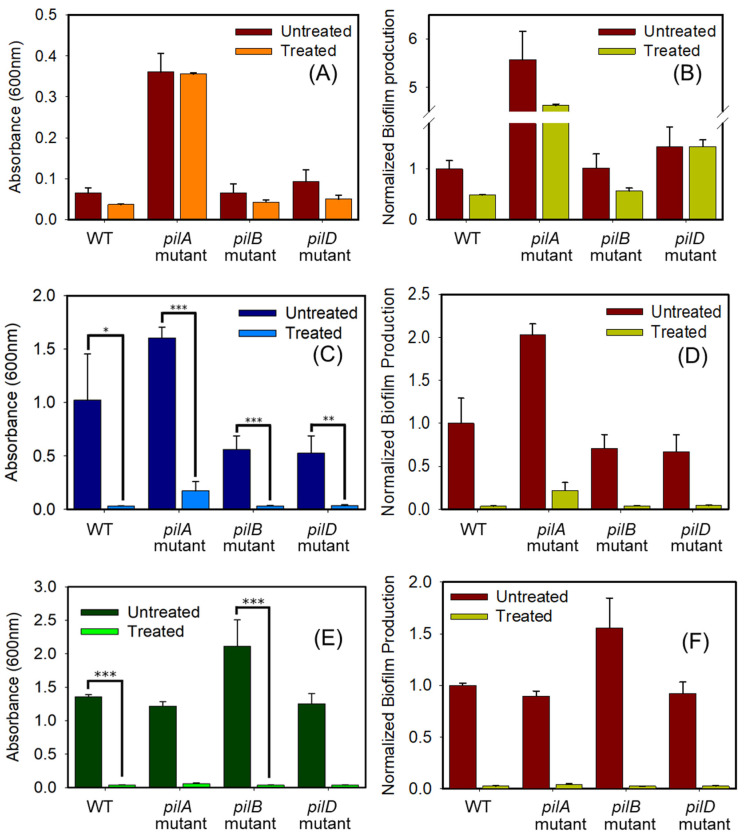
Comparison of biofilm formation by the AB5075 strain and its mutants following exposure to 32 µg/mL TAZ for 8, 24, and 36 h, with antibiotic replenishment every 12 h. The figure presents both absolute biofilm values and values normalized to the untreated WT strain. (**A**,**B**) Biofilm formation after 8 h of TAZ exposure (note: normalized results involve scaling adjustments due to high values obtained for the *pilA* mutant); (**C**,**D**) after 24 h of exposure; and (**E**,**F**) after 36 h of exposure. * indicates significance (*p* < 0.01), ** indicates significance (*p* = 0.006), *** indicates significance (*p* ≤ 0.001).

Figure 4 presents the biofilm formation results following exposure to 32 µg/mL TAZ for 8, 24, and 36 h, with antibiotic replenishment performed every 12 h. After 8 h of exposure, both the absolute biofilm values (Figure 4A) and those normalized to the untreated WT strain (Figure 4B) showed no statistically significant differences between the TAZ-treated and untreated groups, indicating that short-term antibiotic exposure had no measurable impact on biofilm formation. At this early time point, TAZ appears to exert limited inhibitory impact on *A. baumannii* biofilm development. This observation aligns with previous reports highlighting the ability of antibiotic-resistant pathogens to tolerate antimicrobial exposure over short durations [53]. It is therefore possible that AB5075 possesses adaptive mechanisms that sustain biofilm production during initial phases of antibiotic stress, a phenomenon noted in the literature [10,54]. As shown in Figure 4B, the *pilA* mutant exhibited the highest level of biofilm production, whereas the *pilD* and *pilB* mutants displayed levels comparable to the parental strain. These results suggest that disruption of specific T4P-related genes leads to variable effects on biofilm formation.

The findings from our experiment assessing biofilm formation over a 24 h period, depicted in Figure 4C,D, revealed a marked difference in biofilm production between strains exposed to TAZ and those left untreated. Continuous exposure to 32 µg/mL TAZ, with replenishment every 12 h, appeared to significantly impact biofilm formation dynamics over time. While initial results at the 8 h mark showed minimal differences, suggesting a degree of short-term antibiotic tolerance, the data at 24 h revealed a cumulative inhibitory effect on biofilm production. These findings suggest that AB5075 strains, including those with T4P mutations, become increasingly susceptible upon prolonged TAZ exposure. As illustrated in the normalized data (Figure 4B), all mutant strains (*pilA, pilB*, and *pilD* mutants) produced biofilms at levels equal to or greater than the WT at 8 h. However, this trend shifted in the 24 h normalized results (Figure 4D), where mutant strains displayed reduced biofilm formation relative to the WT. These findings suggest that while T4P-deficient mutants exhibit accelerated biofilm production in the early stages, they may fail to maintain or further develop mature biofilms over extended periods. In contrast, the WT strain, which showed minimal biofilm formation at 8 h, exhibited a substantial increase by 24 h, consistent with previous reports indicating that 24 h is sufficient for biofilm maturation [55]. This temporal shift in biofilm kinetics implies that the WT strain may follow a more gradual but sustained biofilm development trajectory, whereas mutant strains display an early but transient response that may not support long-term biofilm stability. Although AB5075 is a clinically relevant and well-characterized *A. baumannii* strain, reliance on a single genetic background limits the generalizability of our findings. Future studies using multiple strain backgrounds are needed to evaluate whether the observed phenotypes are conserved.

To further validate our observations following the 24 h exposure period, the strains were subjected to 32 µg/mL TAZ for an extended duration of 36 h, with media replenishment every 12 h. As shown in Figure 4E, biofilm formation continued to increase in the untreated groups for the WT, *pilB* mutant, and *pilD* mutant strains, with the exception of the untreated *pilA* mutant strain. In contrast, continuous TAZ exposure for 36 h resulted in a marked reduction in biofilm formation across all strains, with no exceptions (Figure 4E,F). However, complete biofilm eradication was not achieved, suggesting that even longer exposure may be necessary for full inhibition.

### 2.5. Tazobactam Inhibition of Biofilms Formed by the AB5075 Strain and Its Mutants

**Figure 5:**
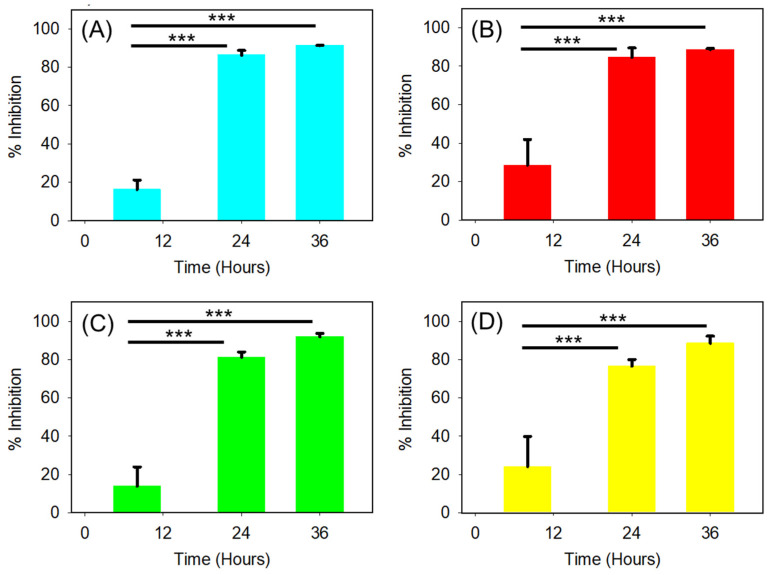
Comparison of percentage inhibition of biofilms formed by the AB5075 strain and its mutants following exposure to 32 µg/mL TAZ for 8, 24, and 36 h, with antibiotic replenishment every 12 h. (**A**) WT; (**B**) *pilA* mutant; (**C**) *pilB* mutant; and (**D**) *pilD* mutant. *** indicates significance (*p* ≤ 0.001).

The data presented in Figure 4 were analyzed to determine the percentages of biofilm inhibition after exposure to 32 µg/mL TAZ for 8, 24, and 36 h. Biofilm inhibition percentages were calculated by comparing the biofilm amount at each time point to that measured without TAZ treatment. As mentioned previously, after 8 h of TAZ exposure, both the absolute biofilm values (Figure 4A) and those normalized to the untreated WT strain (Figure 4B) showed no statistically significant differences, indicating no measurable biofilm inhibition at this early stage. However, as shown in Figure 5A, TAZ treatment of the WT strain led to 86% inhibition at 24 h, which increased slightly to 89% by 36 h. The *pilA* mutant (Figure 5B) exhibited 76% inhibition at 24 h and 82% at 36 h. Similarly, the *pilB* mutant (Figure 5C) showed 78% inhibition at 24 h, increasing to 91% by 36 h. Lastly, the *pilD* mutant (Figure 5D) displayed 70% inhibition at 24 h and reached 86% at 36 h. These results confirm that prolonged exposure to TAZ enhances biofilm inhibition in all tested strains, with the extent of inhibition varying based on the genetic background. A clear and consistent trend emerged, demonstrating that biofilm inhibition increased with extended antibiotic exposure. The use of fresh antibiotic-containing medium every 12 h likely contributed to this effect by maintaining inhibitory pressure throughout the incubation period.

## 3. Materials and Methods

### 3.1. Bacterial Strains

The wild-type AB5075 strain and its pili-deficient mutants (*pilA*, *pilB*, and *pilD* mutants) were kindly provided by Dr. Jieh-Juen Yu (Department of Molecular Microbiology and Immunology, University of Texas at San Antonio). These mutants were originally derived from the AB5075 transposon mutant library developed by Gallagher et al. (2015), which was constructed using random mutagenesis with two distinct Tn5-derived transposons carrying different resistance markers [22]. The majority of mutants in the finalized three-allele set were generated using the T26 transposon, which confers tetracycline resistance [22].

### 3.2. Measurement of Planktonic Growth Kinetics

Bacteria were prepared from a culture stock and inoculated into sterile Luria–Bertani (LB) media. Bacterial cultures were grown overnight at 37 °C while shaking at 150 rpm. Once the bacterial culture reached a specific optical density (OD) in the exponential phase, the bacteria were then subcultured into fresh LB media at a 1:4 ratio in triplicates per strain in a 96-well plate. Once in the 96-well plate, absorbances were measured with a BioTek Cytation5 imaging reader (Agilent Technologies, Santa Clara, CA, 95051, USA) every 30 min at a wavelength of 600 nm. After that, Figure 1 was plotted using the measurements of time (hours) versus OD at 600 nm to obtain the growth kinetics curves of the bacterial strains. Figure 1 and Figure S1 were used to determine the specific growth constant (µ) and the doubling time (t_d_) for each bacterial type.

### 3.3. Measurement of Colony Growth Kinetics

The colony forming units per volume (CFUs/mL) were determined at different time points within the exponential phase of growth. Bacteria were grown as described above planktonically, and absorbances were recorded every 30 min until the late exponential phase. Absorbance thresholds were selected to be approximately consistent across all strains, even though the actual time at which each strain reached these OD values differed due to strain-specific growth kinetics. Samples were then taken at four different points within this phase. At each given absorbance, samples were then diluted 1:10 and the process was repeated to produce diluted bacterial concentrations of 10^−4^, 10^−5^, 10^−6^, and 10^−7^. Once the desired concentrations were obtained, 50 μL of each concentration was plated onto tryptic soy agar (TSA) plates in quadrants, with each quadrant representing a different concentration. The plating was done in triplicates and the plates were tilted in cardinal directions to form a plus (+) shape. The plates were then incubated at 37 °C for approximately 15 h without shaking. After incubation, the plates were then counted to determine the CFU/mL. A calibration curve of the absorbance/hour versus the CFUs/mL was constructed and a line was fit to the data to quantify bacterial growth. All growth kinetics were conducted in biological triplicates and the CFU experiments were carried out in technical replicates to ensure the reproducibility and accuracy of the results.

### 3.4. Time–Kill Assays

A time–kill assay is used to test the efficacy of an antimicrobial agent as a function of time [56,57]. The purpose in this study was to evaluate how effectively TAZ, in our case, can kill *A. baumannii*. The time–kill assay was conducted using a 32 µg/mL concentration of TAZ. Briefly, all four strains were grown overnight into a 96-well plate in triplicates within 100 µL of LB and were exposed to 100 µL of a 32 µg/mL concentration of TAZ. Growth kinetics were then quantified using the absorbance at 600 nm as a function of time. CFU/mL were also quantified at three time points (4, 11, and 20 h), as described above. The 32 µg/mL of TAZ was refreshed every 12 h and the experiment was run for 40 h.

### 3.5. Biofilm Formation Assays

Biofilm formation was assessed using the microtiter dish assay as described by O’Toole (2011) [58], with minor modifications. Biofilms were formed by subculturing the bacterial strains in LB media and then growing in a static incubator at 37 °C for approximately 12 h. Since multiple strains were being evaluated, we ensured we adjusted the turbidity (OD600 nm) to approximately 0.060 absorbance in order to standardize all cultures to a consistent cell density before diluting as needed in a 96-well plate. The plates containing samples were incubated for an additional 12 h at 37 °C. After that, the bacteria were then aspirated in the well plate, washed with deionized water (DIW) once, and the crystal violet (CV) (0.1%) solution (5 mL of CV and 45 mL of DIW) was then added. Afterwards, the solution was allowed to interact with the bacterial culture for 10–15 min before aspirating the dye and washing with DIW twice. To properly quantify the biofilm, 30% (*v*/*v*) of acetic acid was added to the wells and allowed to stand for 5 min before determining the absorbance at 550 nm in the BioTek Cytation5 imaging reader.

A concentration of 32 µg/mL TAZ was applied in order to observe differences between treated and untreated strains and measure the difference in biofilm production. Briefly, the bacterial strains were cultured overnight for approximately 17 h, and then adjusted to an absorbance similar to the exponential phase absorbance determined from previous protocols. From then, the adjusted culture was diluted as 100 μL into 9.9 mL of LB media, and then 100 μL of culture was placed into a 96-well plate in triplicate along with the antibiotic. A total of 200 μL of the same culture was also placed into the 96-well plate as the positive control. Wells that contained LB medium alone or TAZ alone were also measured in triplicates as negative controls.

When it came to antibiotic treatments, once all the wells were prepared, they were grown to the desired time at 37 °C without shaking the plates. For the plates examined at 24 and 36 h, the treated wells were refreshed with antibiotic-infused LB media and the untreated wells served as a positive control with pure LB media. This was done to both mitigate desiccation in the wells and to ensure the bacteria were constantly exposed to the same antibiotic concentration, assuming regular nutrient uptake was occurring. The wells used as negative controls also had CV and acetic acid and were used as the blank for the crystal violet readings. The percentage biofilm inhibition was quantified as follows:

$$\mathrm{Biofilm}\;\mathrm{inhibition}\;\%\;=\;\frac{\mathrm{Untreated}\;\mathrm{Strain}-\mathrm{Treated}\;\mathrm{Strain}}{\mathrm{Untreated}\;\mathrm{Strain}}\times 100\;$$

Biofilm formation was assessed at 8, 24, and 36 h.

### 3.6. Statistical Analysis

Results from this study were assessed via different statistical methods. The one-sample *t*-test was used to compare data that belongs to a single strain, such as absorbances measured from the biofilm assay, provided it passed the normality Shapiro–Wilk Test. When comparing non-parametric group comparisons, such as mutant–WT comparisons or WT–WT time-dependent comparisons, the Mann–Whitney Rank Sum Test was used. If multiple groups were compared, the Kruskal–Wallis One Way Analysis of Variance (ANOVA) method was used. When all pairwise comparisons and comparisons against a control group following rank-based ANOVA were conducted, Dunn’s test was used. Data compared were considered statistically significantly different when *p* values were less than 0.05.

## 4. Conclusions

Our findings revealed distinct phenotypic consequences of T4P disruption. The *pilA* mutant exhibited accelerated planktonic growth rate compared to the WT strain. Colony formation assays consistently demonstrated enhanced CFU/mL counts for the *pilA* mutant, likely due to impaired twitching motility and redirection of energy toward biomass accumulation. Time–kill assays showed that 32 µg/mL of TAZ suppressed all strains over time. However, the *pilD* mutant displayed transient tolerance at an early stage of exposure, evident in both the higher absorbance and CFU/mL counts. The *pilA* mutant exhibited a similar response even earlier, but only in CFU measurements, suggesting distinct adaptive mechanisms. In terms of biofilm formation, the *pilA* mutant showed the highest early biofilm biomass, likely as a result of immobilization and energy redirection. Prolonged TAZ exposure, however, led to marked inhibition of biofilm development in all strains. Initially, at 8 h, minimal differences were observed, suggesting a degree of short-term tolerance, but data at 24 and 36 h revealed a cumulative and enhanced inhibitory effect on biofilm production. These results suggest that the roles of T4P components in *A. baumannii* are highly context-dependent and strain-specific, particularly regarding biofilm dynamics and antibiotic responsiveness. The divergent phenotypes observed in pili-deficient mutants indicate that T4P disruption can lead to compensatory physiological shifts, including altered growth strategies, persistence traits, and differential surface behavior.

Our findings also contribute additional evidence that TAZ, beyond its classical role as a β-lactamase inhibitor, exhibits intrinsic antibacterial activity against the highly virulent AB5075 strain and its mutants, supporting its potential repurposing as a standalone therapy for biofilm-associated infections. Further investigations are needed to elucidate the molecular mechanisms underlying T4P-mediated tolerance and persistence, particularly regarding PilA and PilD and their interactions with other membrane-associated systems. Long-term exposure models, transcriptomic analyses, and mutant complementation studies will be valuable to better define the regulatory networks involved.

In conclusion, our findings offer phenotypic insights into how *pilA*, *pilB*, and *pilD* influence growth, biofilm formation, and antibiotic response in *A. baumannii* 5075. Although these observations are limited to a single genetic background and do not resolve the underlying molecular mechanisms, they provide a valuable foundation for future mechanistic studies aimed at elucidating the pathways through which these genes modulate bacterial physiology and antibiotic tolerance. Taken together, these findings highlight both the complexity of bacterial adaptation and the potential therapeutic relevance of reevaluating agents such as TAZ.

## Figures and Tables

**Table 1:** Specific growth constants (μ, h^−1^) and doubling times (t_d_, min) of the AB5075 WT and mutant strains calculated from the slopes of linear regressions on the natural logarithm of optical density values (OD600 nm) over two time intervals (2–5 h and 5–8 h), representing the early and mid-exponential phases, respectively. Specific growth constants were derived from the slopes of ln(OD600) vs. time plots, and doubling times were computed as t_d_ = ln(2)/μ.

Strain	Time Interval: 2–5 h		Time Interval: 5–8 h	
	µ (h^−1^)	t_d_ (min)	µ (h^−1^)	t_d_ (min)
AB5075 WT	0.348	119.4	0.271	153.5
AB5075 *pilA* mutant	0.974	42.7	0.311	134.0
AB5075 *pilB* mutant	0.558	74.5	0.200	208.0
AB5075 *pilD* mutant	0.539	77.2	0.185	225.0

**Table 2:** A summary of the colony formation unit (CFU/mL) counts and corresponding absorbance values and time points for each tested AB5075 strain.

	Time Point 1			Time Point 2			Time Point 3			Time Point 4		
Strain	CFU/mL	Abs	Time (h)	CFU/mL	Abs	Time (h)	CFU/mL	Abs	Time (h)	CFU/mL	Abs	Time (h)
AB5075 WT	1.47 × 10^7^	0.112	1.9	4.83 × 10^7^	0.311	4.7	6.65 × 10^7^	0.500	6.3	8.60 × 10^7^	0.746	7.9
AB5075 *pilA* mutant	2.15 × 10^8^	0.189	4.6	3.03 × 10^8^	0.325	5.8	4.32 × 10^8^	0.455	7.8	5.87 × 10^8^	0.610	8.4
AB5075 *pilB* mutant	6.14 × 10^7^	0.201	2.7	8.47 × 10^7^	0.284	3.3	1.25 × 10^8^	0.398	4.0	1.68 × 10^8^	0.484	4.3
AB5075 *pilD* mutant	6.20 × 10^7^	0.227	2.7	1.33 × 10^8^	0.391	3.6	3.33 × 10^8^	0.581	4.7	3.98 × 10^8^	0.770	5.6

## Data Availability

Data availability is restricted due to ongoing research efforts and possible disclosures.

## Supplementary Materials

The following supporting information can be downloaded at https://www.mdpi.com/article/10.3390/antibiotics14080816/s1: Figure S1: (A) Plot of the natural logarithm of average OD_600_ measurements for AB5075 WT and mutant strains over time, with each strain depicted using a unique color and symbol. (B) Linear regressions on the natural logarithm of OD_600_ values over two time intervals, 2–5 h and 5–8 h, representing the early and mid-exponential phases, respectively. The slopes of these regression lines correspond to the specific growth rates (μ), which were subsequently used to calculate doubling times. The average R^2^ values for the regressions were 0.981 ± 0.014 (early-exponential phase) and 0.982 ± 0.012 (mid-exponential phase), indicating strong linearity within the selected intervals. Figure S2: Time–kill assay based on absorbance measurements of the AB5075 strain and its mutants treated with 32 µg/mL TAZ, with antibiotic replenishment every 12 h, conducted over a period of approximately 40 h. Figure S3: Time–kill assay based on CFU/mL measurements of the AB5075 strain and its mutants treated with 32 µg/mL TAZ, with antibiotic replenishment every 12 h, conducted at different time points (4, 11, and 20 h referring to time points 1, 2, and 3, respectively) for the (A) WT; (B) *pilA* mutant; (C) *pilB* mutant; and (D) *pilD* mutant. ** Indicates significance (*p* < 0.05).
