# Supplementary material for: Phenotypic Characterization of pilA, pilB, and pilD Mutants of Acinetobacter baumannii 5075: Impacts on Growth, Biofilm Formation, and Tazobactam Response
Source: Antibiotics (Basel). 2025 Aug 9;14(8):816. doi: 10.3390/antibiotics14080816 (PMC12382802; doi:10.3390/antibiotics14080816)
Supplement: Supplementary file 1 [file antibiotics-14-00816-s001.zip › antibiotics-3768039-supplementary.pdf]

Supporting Information for:

Phenotypic Characterization of *pilA*, *pilB*, and *pilD* Mutants of *Acinetobacter baumannii* 5075: Impacts on Growth, Biofilm Formation, and Tazobactam Response

Joel H. Salinas, Jr., Fatma Pinar Gordesli-Duatepe, Angelica Diaz-Sanchez and Nehal I. Abu-Lail *

*Correspondence: nehal.abu-lail@utsa.edu; Tel.: +1-210-458-8131

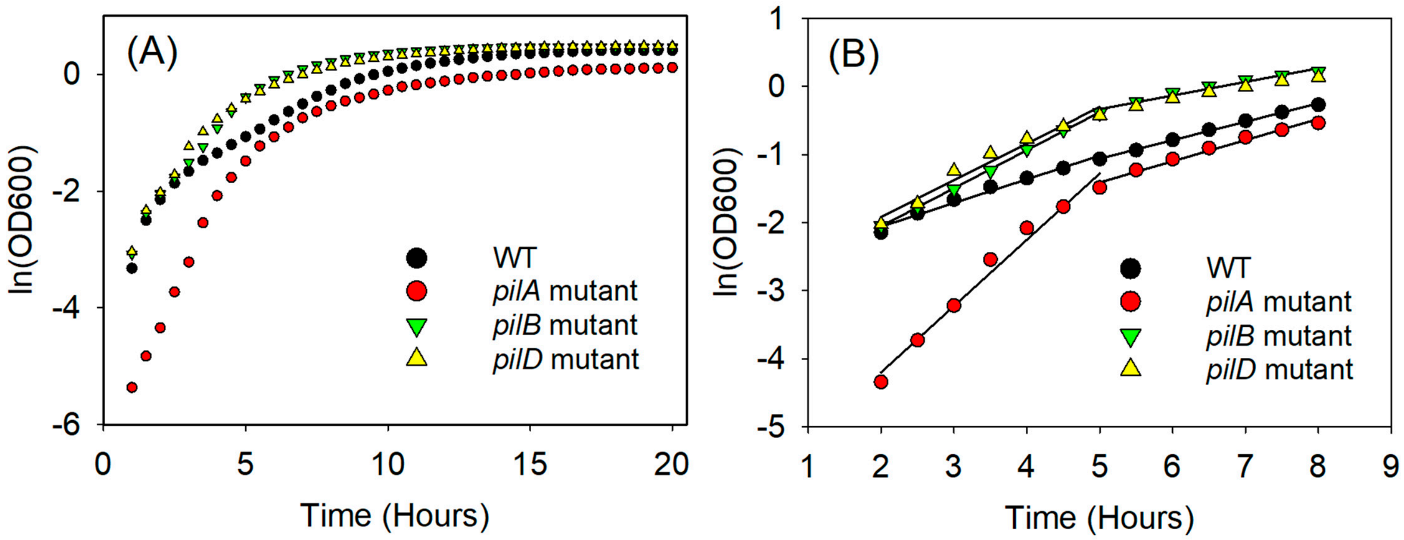

Figure S1. (A) Plot of the natural logarithm of average OD_{600} measurements for AB5075 WT and mutant strains over time, with each strain depicted using a unique color and symbol. (B) Linear regressions on the natural logarithm of OD_{600} values over two time intervals: 2–5 h and 5–8 h, representing the early and mid-exponential phases, respectively. The slopes of these regression lines correspond to the specific growth rates (μ), which were subsequently used to calculate doubling times. The average R^2 values for the regressions were 0.981 ± 0.014 (early-exponential phase) and 0.982 ± 0.012 (mid-exponential phase), indicating strong linearity within the selected intervals.

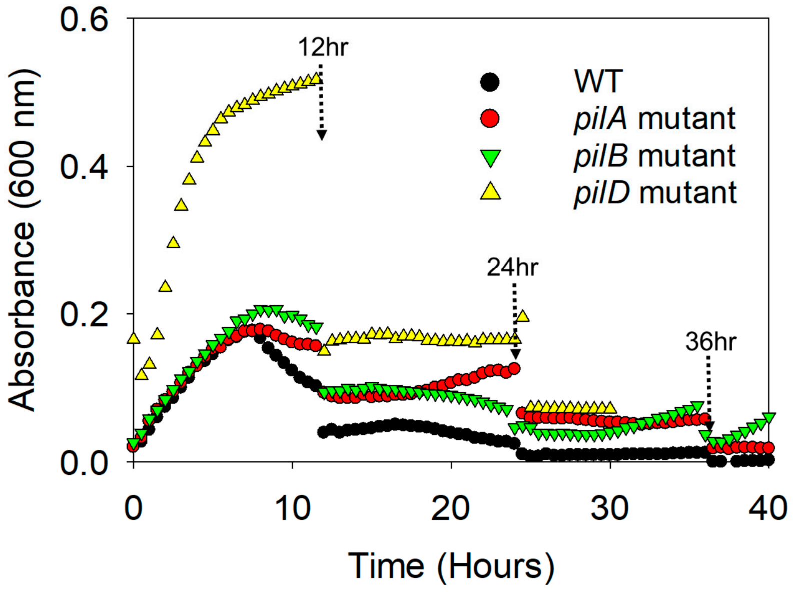

Figure S2. Time-kill assay based on absorbance measurements of AB5075 strain and its mutants treated with 32 μg/mL TAZ, with antibiotic replenishment every 12 h, conducted over a period of approximately 40 h.

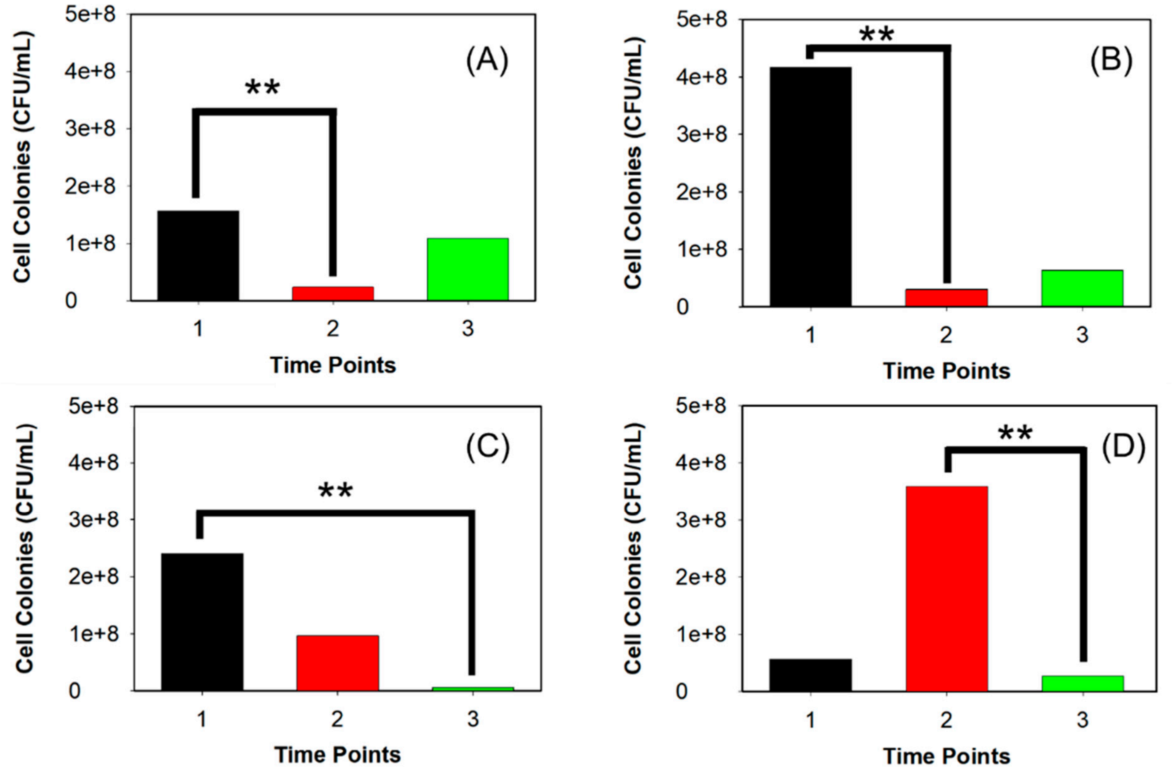

Figure S3. Time-kill assay based on CFU/mL measurements of AB5075 strain and its mutants treated with 32 μg/mL TAZ, with antibiotic replenishment every 12 h, conducted at different time points (4, 11, and 20 h referring to time points 1, 2, and 3 respectively) for: (A) WT; (B) *pilA* mutant; (C) *pilB* mutant; (D) *pilD* mutant. ** Indicates significance (p<0.05).
